# Clinical validation of PEA-driven Olink proteomic discovery: INPP1 and ARHGAP25 serum biomarkers improve early breast cancer diagnosis

**DOI:** 10.1016/j.tranon.2026.102804

**Published:** 2026-05-04

**Authors:** Jingchun Huang, Lixin Gao, Liliia Glazutdinova, Xuyao Ji, Jing Zhang, Youmei Lu, Siyao Zhang, Yu Liu

**Affiliations:** a Department of Laboratory Medicine, Harbin Medical University Affiliated Fourth Hospital, Harbin, Heilongjiang 150000, China; b Department of Laboratory Medicine, The 960th Hospital of the People’s Liberation Army, Jinan, Shandong 250000, China

**Keywords:** Olink, Proteomics, Breast cancer, Biomarkers, INPP1, ARHGAP25

## Abstract

**Background:**

Breast cancer is the most common malignant tumor in women all over the world, accounting for 15% of all female cancer-related mortality. Due to the limited sensitivity of current serum biomarkers (such as CA15–3, CEA) and the invasiveness of imaging/biopsy methods, early detection of breast cancer is still challenging. The purpose of this study is to use Olink proteomics to identify new diagnostic markers of breast cancer and integrate them into a multi-protein diagnostic model to improve the accuracy of early detection.

**Methods:**

Serum proteomic profiling was performed via Olink’s proximity extension assay (PEA) in a discovery cohort (15 breast cancer patients vs. 16 healthy controls). Differentially expressed proteins were analyzed to construct a diagnostic model, which was validated in an independent cohort (111 breast cancer patients [56 early-stage, 55 late-stage] vs. 95 healthy controls).

**Results:**

The combination of INPP1 (first reported as downregulated in breast cancer serum) and ARHGAP25 demonstrated high diagnostic accuracy, achieving AUCs of 0.8458 (discovery cohort) and 0.8506 (validation cohort). Notably, the model retained efficacy in early-stage detection (AUC = 0.7598).

**Conclusion:**

This study identifies a novel serum protein panel (INPP1/ARHGAP25) as a minimally invasive tool for breast cancer diagnosis, particularly valuable for early-stage screening. The findings underscore the potential of proteomics-driven biomarker discovery to address clinical unmet needs.

## Highlights

• First serum biomarker panel combining INPP1 and ARHGAP25 for early breast cancer.
• Utilizes high-throughput Olink PEA proteomic technology for biomarker discovery.
• Robust diagnostic performance validated in an independent cohort (AUC > 0.85).
• Effective for early-stage detection (Stage I-II) with clinical translational potential.
• Establishes a multi-omics framework linking proteomics to genomics and metabolomics.

## Introduction

According to data from GLOBOCAN 2020, breast cancer has surpassed lung cancer as the malignant tumor with the highest incidence in women, accounting for 24.5% (2.3 million cases) of newly diagnosed cancers in women worldwide. However, a World Health Organization report indicates that its early diagnosis rate is <30% [1]. This is still a huge challenge in the global health goal. The complex pathogenesis and diverse clinical manifestations of breast cancer pose significant challenges to its prevention and diagnosis [2]. Although the prognosis of breast cancer patients has improved in recent decades due to the popularity of screening, there are still significant limitations in the accuracy of early diagnosis, treatment, and prognosis prediction due to the lack of large-scale screening, early detection, and dynamic, rapid detection of tumors [3]. Although the sensitivity of common clinical serum markers CA15–3 and CEA in advanced breast cancer can reach 75% −90%, their efficacy in early diagnosis is not clear, and they are not recommended as early diagnosis markers [4]. Therefore, it is urgent to develop new diagnostic markers with high sensitivity and non-invasive.

In recent years, liquid biopsy technology has developed rapidly. This technology enables molecular analysis of liquid samples (primarily blood). As a non-invasive diagnosis and monitoring tool, it has shown great potential in the fields of early screening, efficacy evaluation, recurrence monitoring, and so on, and promoted its routine clinical application in cancer patients [5]. Among them, DNA (circulating tumor DNA) and proteomics are the two core technical routes. Proteins are the direct executors of most cell functions and direct drug targets in most cancer therapies. Proteomics has unique advantages in the identification of new biomarkers because it can directly reflect the functional phenotype, reveal tissue-specific information, and determine the source of tumors [6].

In the post-genome era, Proteomics plays an important role in biomedical research. Unlike the detection bottleneck of traditional proteomics technology, the progress of high-throughput proteomics not only helps to understand the pathogenesis better, identify the characteristic signal network of specific diseases, but also helps to assess the risk of various diseases [7]. Olink is a high-throughput protein detection platform based on PEA (Proximity Extension Assay). Pea can detect proteins with ultra-high sensitivity (sensitivity up to fg/mL level) and specificity by double recognition of target proteins by DNA-labeled antibodies and amplification of signals by PCR. Compared with traditional mass spectrometry technology, PEA can improve the detection sensitivity of low abundance proteins by 2–3 orders of magnitude, and only 1 μL of serum sample is needed to analyze 1500+proteins at the same time, covering many kinds of biomarkers such as inflammation, tumor, metabolism, etc., which solves the problem of clinical precious samples and the limitations of traditional proteomics detection. At the same time, combined with excellent inter-batch repeatability (CV<15%), Olink has become an ideal tool for biomarker research [8,9]. In previous studies, it has also been shown that the Olink proteomics has a great advantage. The selected biomarkers can significantly improve the disease risk stratification of patients, and have ideal predictive performance for a variety of diseases and deaths, even better than the established clinical predictive factors [10].

In this study, we used Olink proteomics to detect the expression levels of tumor-associated proteins in the serum of breast cancer patients and healthy individuals, in order to find new diagnostic markers of breast cancer. The objective is to integrate the strategy of multi-protein diagnostic combination and cross-platform validation (Olink → ELISA) to find and validate new serum markers for early diagnosis of breast cancer.

## Materials and methods

### Study design and participant enrollment

This study employed a prospective specimen collection and a retrospective nested case-control design in accordance with STARD guidelines. And this study was conducted in accordance with the principles of the Declaration of Helsinki.

Between May 2023 and June 2024, we consecutively recruited adult women presenting at the Fourth Affiliated Hospital of Harbin Medical University with breast lesions suspicious for malignancy and scheduled for core needle biopsy or diagnostic surgery.

Following pathological examination, a consecutive series of participants with confirmed breast cancer was included in the case group. Individuals whose pathological results revealed benign breast diseases (e.g., fibroadenoma, cysts) were excluded from this particular analysis. The healthy control group consisted of age-matched volunteers recruited from routine health screenings during the same period, with no history of any breast disease or malignancy.

### Study cohorts

The discovery cohort comprised 15 newly diagnosed breast cancer patients (9 early-stage, 6 late-stage; 10 Luminal, 3 HER-2+, 2 TNBC (Triple-Negative Breast Cancer)) and 16 age/sex-matched healthy controls; The validation cohort included 111 patients with breast cancer (56 in Early stage, 55 in Late stage; 70 Luminal, 29 HER-2+, 12 TNBC) and 95 healthy controls. The diagnosis and staging of all cases were in accordance with the guidelines and specifications for breast cancer diagnosis and treatment of the China Anti-Cancer Association (2024 version) [11].

In the ROC curve of marker screening, 16 cases of liver cancer, lung cancer, colorectal cancer, and rheumatoid arthritis were included in the new control group.

The study protocol was approved by the Ethics Committee of the Fourth Affiliated Hospital of Harbin Medical University, China (approval No KY-2022–043). All participants provided written informed consent.

### Plasma sample collection

Fasting venous blood (4–5 mL) was collected into coagulation tubes. Serum was separated by centrifugation at 3000 rpm for 5 min, aliquoted, and stored at −80 °C until analysis. Exclude unqualified samples such as hemolysis, fatty blood, and jaundice.

### Olink proteome detection

Serum samples from breast cancer patients and healthy controls were analyzed using the Olink Explore Oncology III panel. This assay is based on the Proximity Extension Assay (PEA) technology, where paired oligonucleotide-labeled antibodies enable highly specific protein detection [12]. The workflow, technical principle and data processing of Olink proteomics are described in the research paper of Sun et al. [13] Olink assays were performed in triplicate, and batch effects were adjusted using the ComBat algorithm [14].

### ELISA validation

In the later cross-platform verification, we used ELISA to detect the verification queue. The plasma samples of 111 patients with breast cancer (56 cases in the early stage, 55 cases in the late stage) and 95 healthy controls were included. Human inositol polyphosphate 1-phosphatase (INPP1) and human Rho GTPase activator protein (ARHGAP25) ELISA kits were used for analysis and detection (China Ruixin Biology). The detection methods and data analysis were carried out in strict accordance with the manufacturer's scheme.

### Blinding procedure

The laboratory personnel who performed the Olink PEA assays and the data scientists who conducted the subsequent statistical analysis were completely blinded to the clinical information and reference standard (pathological) results of all samples. The pathologists who made the definitive pathological diagnoses were blinded to the results of the Olink proteomic analysis and the group allocation of the samples during their assessment.

### Data analysis

For the obtained Olink data, the normalized protein expression (NPX) value was analyzed by Olink analysis [15]. Differentially expressed proteins (DEPs) were defined as those with an FDR-adjusted p-value < 0.05. Volcano plots, heatmaps, and Gene Ontology (GO)/KEGG enrichment analyses were generated using the R package ggplot2 [16] In addition, the ROCR software package was used to create a receiver operating characteristic (ROC) curve [17]. For the ELISA results, SPSS 25.0 software was used to analyze the data and statistics, and GraphPad Prism 10.1.2 was used to plot. Normally distributed data were analyzed using the Independent Samples *t*-test, while non-normally distributed data were assessed with the Mann-Whitney U test. Spearman’s correlation analysis was used to evaluate the correlation between INPP1/ ARHGAP25 and Ki-67. The sample size for the validation cohort was estimated a priori using a formal power calculation ($\left[\mu \alpha^{2}\times p\times \;\left(1-p\right)\right]/\sigma^{2}$) for the area under the receiver operating characteristic curve (AUC).

## Results

### Basic characteristics of the research object

**Fig. 1:**
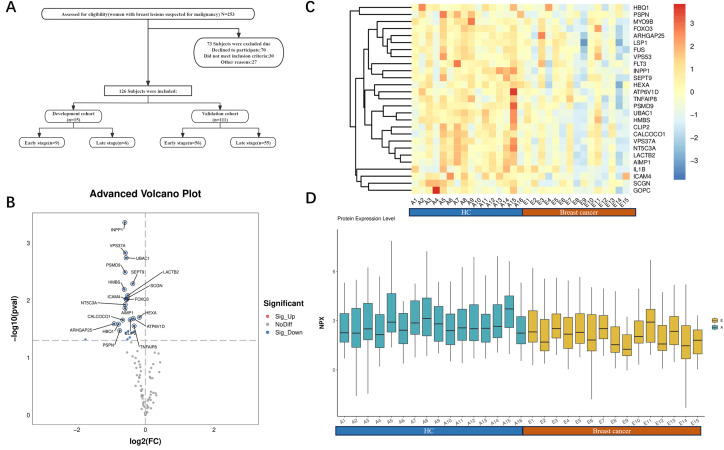
Participant flowchart and all differentially expressed tumor-associated proteins between breast cancer and healthy controls were analyzed by the Olink Oncology panel Ⅲ. (A) Flowchart of participant enrollment, exclusion, and allocation into the discovery and validation cohorts. The primary reason for exclusion after diagnosis was prior treatment. (B) Volcanic map of differential expression of 92 tumor proteins in the Olink panel. Blue dots indicate 27 proteins that were significantly downregulated (p < 0.05). (C) Heatmap showing hierarchical clustering of 27 differentially expressed proteins. (D) Box plots comparing normalized protein expression (NPX) values between groups.

As shown in Table 1, according to the research protocol, the Olink discovery cohort included 31 subjects, including 15 breast cancer patients, 9 in the early stage, and 6 in the late stage, with a mean age of 55.47 ± 11.71; healthy controls had a mean age of 47.31 ± 12.52 years. The ELISA validation cohort included 207 subjects, 111 patients with breast cancer, 56 in the early stage, and 55 in the late stage, with an age of 55.47 ± 10.11; The age of 95 healthy controls was 50.11 ± 11.36. There were no significant differences in age, height, and weight (p > 0.05). The flow of participants through the various stages of the study is detailed in Fig. 1A.

**Table 1:** Basic clinical characteristics of the discovery cohort and the validation cohort.

	Breast cancer group	control group	P value
	Olink cohort		
sample size	N = 15	N = 16	
stage,n(%)			
Early stage	9(60)	NA	
Late stage	6(40)	NA	
types,n(%)			
Luminal	10(66.7)	NA	
Her-2+	3(20)	NA	
TNBC	2(13.3)	NA	
KI-67(%)	50(40∼60)	NA	
Age(years)	55.47±11.71	47.31±12.52	0.072
Height(cm)	161.60±5.37	159.58±5.28	0.3
Weight(kg)	63.93±8.31	58.13±6.14	0.034
	Elisa cohort		
sample size	N = 111	N = 96	
stage,n(%)			
Early stage	56(50.45)	NA	
Late stage	55(49.55)	NA	
types, n(%)			
Luminal	70(63.1)	NA	
Her-2+	29(26.1)	NA	
TNBC	12(10.8)	NA	
KI-67(%)	42.67±20.17	NA	
Age(years)	55.47±10.11	50.11±11.36	0.061
Height(cm)	160.82±4.39	160.26±5.38	0.407
Weight(kg)	60.71±5.61	59.58±6.14	0.168

Early stage:0,Ⅰ,Ⅱ;Late stage:Ⅲ,Ⅳ.

### Olink analysis of tumor differential proteins in breast cancer

Olink proteomics was used to compare the expression levels of 92 tumor-associated proteins between the breast cancer group and healthy controls. Twenty-seven differentially expressed proteins (DEPs) were identified, all of which were downregulated. The volcano plot (Fig. 1B) highlights 27 downregulated DEPs, while the heatmap (Fig. 1C) illustrates their expression trends across individual samples. The box plot (Fig. 1D) displays the normalized protein expression (NPX) values in both groups.

### Analysis of differentially expressed proteins in breast cancer and healthy controls

**Fig. 2:**
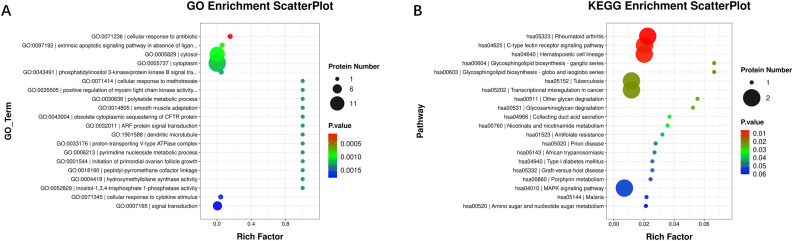
Gene Ontology (GO) and Kyoto Encyclopedia of Genes and Genomes (KEGG) enriched and analyzed the differentially expressed tumor-related proteins. (A) GO function enrichment analysis revealed the top 20 significantly enriched GO terms. (B) KEGG pathway analysis identified the top 20 enriched KEGG pathways.

Gene Ontology (GO) functional enrichment and Kyoto Encyclopedia of Genes and Genomes (KEGG) pathway analyses were conducted for the 27 DEPs. The GO function enrichment analysis (Fig. 2A) revealed that the top 20 enriched terms were primarily associated with signal transduction, cytoplasmic localization, and intracellular processes. KEGG pathway analysis (Fig. 2B) identified the top 20 enriched pathways, including the C-type lectin receptor (CLR) signaling pathway, rheumatoid arthritis-associated pathways, and regulation of hematopoietic cells.

### Screening of breast cancer candidate markers

**Fig. 3:**
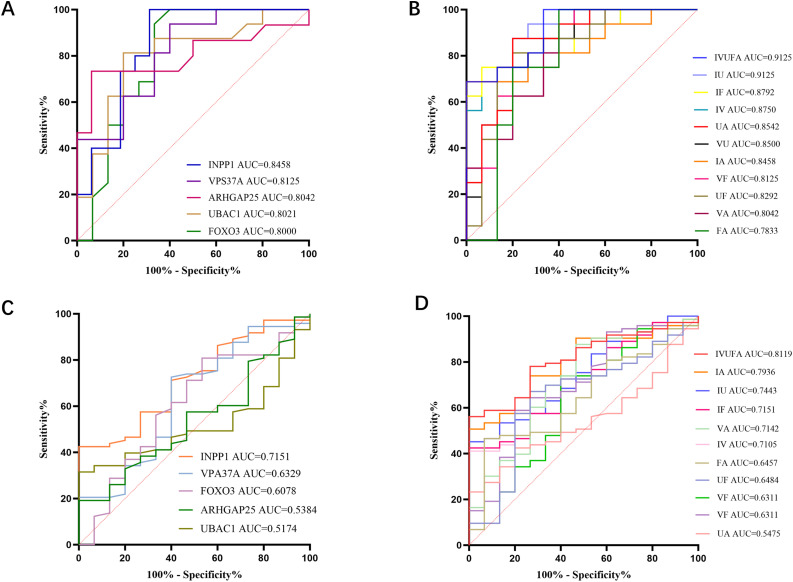
Diagnostic performance of candidate biomarkers for breast cancer. (A) ROC curves of the top five differentially expressed proteins in breast cancer vs. healthy controls. (B) ROC curve of the multi-protein panel in breast cancer vs. healthy controls. (C) ROC curve of the top five differentially expressed proteins in breast cancer vs. other diseases. (D) ROC curve of the INPP1/ARHGAP25 combination in breast cancer vs. other diseases.

ROC analysis was performed for the top five statistically significant proteins identified by Olink, and shows the individual AUC values for each protein (Fig. 3A). Univariate logistic regression analysis was performed to construct a multi-protein diagnostic combination. In the breast cancer and healthy control groups, the AUC values for five protein combinations and for INPP1/UBAC1 protein combinations were both 0.9125 (Fig. 3B). Then, in order to select breast cancer-specific proteins, we compared breast cancer with the control group of other diseases(16 cases of liver cancer, lung cancer, colorectal cancer, and rheumatoid arthritis)(Fig. 3C), in which the AUC values of the five protein combinations reached 0.8119. It is worth noting that the AUC values of INPP1 and ARHGAP25 protein combinations reached 0.7936 (Fig. 3D). Therefore, we prioritized the INPP1/ARHGAP25 combination as the final candidate biomarker pair. This decision was based on its superior ability to distinguish breast cancer from other diseases, as evidenced by its higher sensitivity against the “other disease” control group (0.7936) compared to the INPP1/UBAC1 combination (0.7443). We reason that a biomarker with enhanced recognition of confounding diseases is crucial for reducing misdiagnosis and is better suited for the complex setting of clinical differential diagnosis.

### ELISA verification of INPP1 and ARHGAP25

**Fig. 4:**
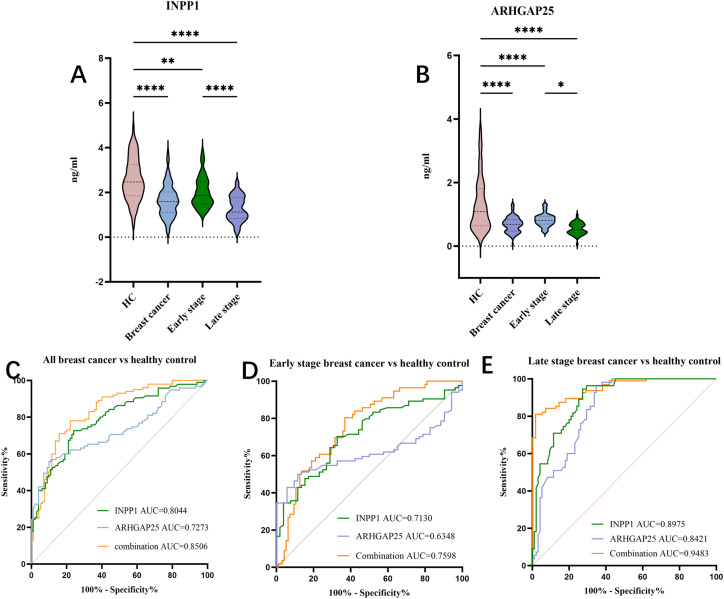
The expression of INPP1 and ARHGAP25 in breast cancer serum was detected by ELISA, and its diagnostic performance was analyzed. (A) INPP1 levels in early-stage, late-stage breast cancer, and healthy controls. (B) ARHGAP25 levels in early-stage, late-stage breast cancer, and healthy controls. (C) ROC curves of INPP1 and ARHGAP25 in overall breast cancer. (D) ROC curves in early-stage breast cancer. (E) ROC curves in late-stage breast cancer. Data are presented as mean ± SD;(*P < 0.05, **P < 0.01, ****P < 0.0001.).

According to the optimal protein diagnostic combination, we identified INPP1 and ARHGAP25 as candidate markers for subsequent experiments, and analysed the levels of INPP1 and ARHGAP25 in different stages of breast cancer by ELISA. ELISA was preformed on 111 plasma samples from patients with breast cancer (56 cases in the early stage and 55 cases in the late stage) and 95 healthy controls. ELISA showed that INPP1 and ARHGAP25 were downregulated in breast cancer patients compared to controls, and compared with the early breast cancer, with INPP1 and ARHGAP25 being significantly downregulated in the advanced breast cancer (Fig. 4A, B), these results are consistent with Olink results (Fig. 1). Based on the ROC curve analysis of INPP1 and ARHGAP25 in breast cancer serum, the AUC of INPP1 was 0.8044 (95% CI:0.7449 to 0.8639, p < 0.0001), The optimal cutoff value (determined by Youden’s index) was >2.08, the sensitivity and specificity were 78.4% and 70.5%, respectively, and the Youden index was 0.489 (Fig. 4C); The AUC of ARHGAP25 was 0.7273 (95%CI:0.6546 to 0.7999, p < 0.0001), the best cut-off value based on Youden index was>0.99, the sensitivity and specificity were 90.1% and 56.8%, respectively, and the Youden index was 0.469 (Fig. 4C). In addition, in the ROC of early breast cancer, the AUC of INPP1 was 0.7130 (95%CI:0.6310 to 0.7949, p < 0.0001), the best cut-off value based on Youden index was>2.05, the sensitivity and specificity were 62.5% and 72.6%, respectively, and the Youden index was 0.351 (Fig. 4D); The AUC of ARHGAP25 was 0.6348 (95% CI: 0.5472 to 0.7224, p = 0.0057), the best cut-off value based on Youden index was>1.00, the sensitivity and specificity were 82.1% and 55.8%, respectively, and the Youden index was 0.379 (Fig. 4D). In the ROC of advanced breast cancer, INPP1 yielded an AUC of 0.8975 (95%CI: 0.8497 to 0.9453, p < 0.0001), the best cut-off value based on Youden index was >2.066, the sensitivity and specificity were 94.5% and 72.6%, respectively, and the Youden index was 0.672 (Fig. 4E); For ARHGAP25, the AUC was 0.8421(95% CI: 0.7814 to 0.9028, p < 0.0001), the best cut-off value based on Youden index was>0.8076, the sensitivity and specificity were 96.4% and 65.3%, respectively, and the Youden index was 0.616(Fig. 4E).

### Diagnostic performance of INPP1 and ARHGAP25 protein models in breast cancer

The combined model of INPP1 and ARHGAP25 achieved an AUC of 0.846 (95% CI: 0.704–0.987, p < 0.01) in the Olink discovery cohort (Fig. 3B). The performance of INPP1 and ARHGAP25 was then validated by ELISA in an independent cohort. The combination of INPP1 and ARHGAP25 achieved an AUC of 0.8506 (95%CI:0.7988 to 0.9025, p < 0.0001) in all breast cancer vs healthy controls, the best cut-off value based on Youden index is>0.65, the sensitivity and specificity are 73.9% and 84.2%, and the Youden index is 0.581 (Fig. 4C); The further analysis shows that the combined AUC in early breast cancer (stage Ⅰ - Ⅱ) can reach 0.7598 (95%CI:0.6829 to 0.8366, p < 0.0001), the best cut-off value based on Youden index is>0.36, and sensitivity and specificity were 80.4% and 63.2%, respectively, and Youden index was 0.436 (Fig. 4D); And the combined AUC in advanced breast cancer (stage Ⅲ- Ⅳ) can reach 0.9483 (95%CI: 0.9176 to 0.9791, p < 0.0001), the best cut-off value based on Youden index is>0.252, and sensitivity and specificity were 81.1% and 98.1%, respectively, and Youden index was 0.792 (Fig. 4E);

### Correlation analysis of INPP1 and ARHGAP25 with breast cancer molecular typing and tumor proliferation activity

**Fig. 5:**
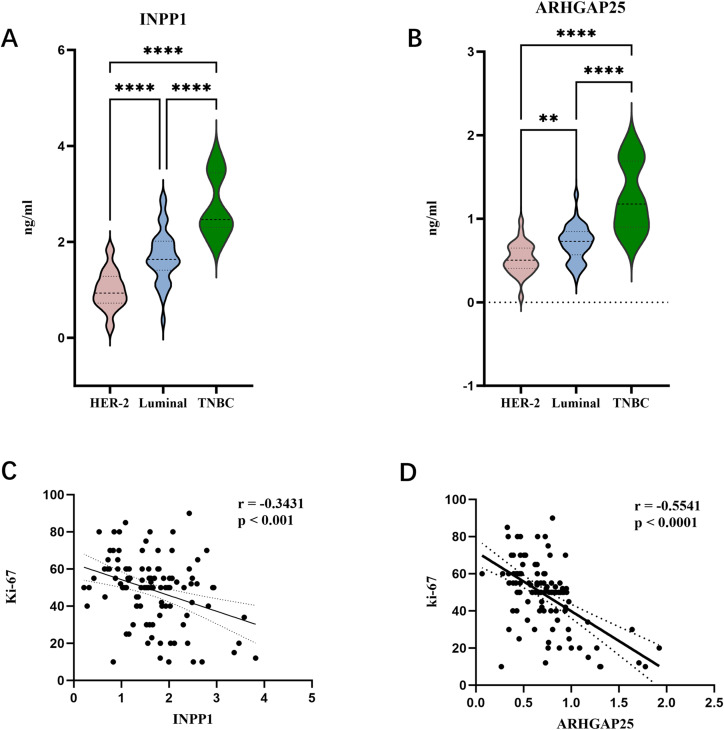
The correlation between breast cancer candidate markers, molecular typing, and Ki-67. (A) The expression level of INPP1 in different molecular subtypes of breast cancer. (B) The expression level of ARHGAP25 in different subtypes of breast cancer. (C) Correlation analysis of INPP1 and Ki-67 (D) Correlation analysis of ARHGAP25 and Ki-67.

INPP1 and ARHGAP25 expression levels were compared across three molecular subtypes of breast cancer. INPP1 and ARHGAP25 expression was significantly lower in HER-2 positive breast cancer than in luminal breast cancer, while the expression of INPP1 and ARHGAP25 was significantly higher in triple negative breast cancer (TNBC) (P < 0.0001) (Fig. 5A, B).INPP1 was moderately negatively correlated with Ki-67 (r=−0.3431, P < 0.001) (Fig. 5C), and ARHGAP25 expression was negatively correlated with Ki-67 proliferation index(r = 0.5541,P < 0.0001) (Fig. 5D).

## Discussion

Breast cancer, with approximately 2.3 million new cases globally in 2022, continues to experience a rising incidence, which is projected to increase by 38% by 2050 [18]. The pathogenesis of breast cancer is a complex process resulting from the interaction among genetic susceptibility, hormone stimulation, abnormal signaling pathways, epigenetic changes, and the microenvironment [19]. From a mechanistic perspective, identifying clinical therapeutic targets and diagnostic markers is crucial, as early diagnosis and treatment can significantly improve patient prognosis and quality of life. However, existing diagnostic markers are suboptimal for early screening, while the incidence of breast cancer continues to increase. Therefore, we need to urgently find new diagnostic markers to improve the early diagnosis rate of breast cancer and reduce the mortality and adverse prognosis of breast cancer.

Protein markers have been widely verified in clinical practice [20]. Unlike traditional proteomic methods, Olink proteomics overcomes their limitations through the sensitive detection of low-abundance proteins. Olink's ultra-high sensitivity and specificity give it distinct advantages in developing novel diagnostic markers.

Using Olink technology, we detected differentially expressed proteins (DEPs) in blood samples from breast cancer patients and healthy controls, identifying 27 DEPs, all of which were downregulated. These DEPs were enriched in tumor-related functions and pathways, including signal transduction, cytoplasmic processes, and the C-type lectin receptor (CLR) signaling pathway. Notably, the CLR pathway—a key pattern recognition receptor (PRR)—plays critical roles in innate immune responses and tumor microenvironment regulation [21,22]. The systemic downregulation of these proteins, particularly within the CLR pathway, suggests a potential state of broad immune regulation or suppression, which may represent a systemic host response to the tumor and contribute to a permissive microenvironment for progression.

Through multi-protein combination screening, we identified INPP1 and ARHGAP25 as a diagnostic pair. Previous studies by Han et al. demonstrated that ARHGAP25 suppresses the proliferation, migration, and invasion of breast cancer cells through the ARHGAP25/Wnt/ASCL2 feedback loop, though their study focused on tissue expression rather than serum levels [23]. INPP1 is involved in carcinogenesis through glycolytic reprogramming. Although previous studies have reported no significant differential expression of INPP1 in primary breast tumor tissues [24], our high-sensitivity Olink proteomic analysis detected a significant decrease in INPP1 protein levels in the serum of breast cancer patients. This circulating protein signature suggests that the release or secretion pattern of INPP1 may differ from its expression pattern within tissues, providing a new dimension for understanding its role in breast cancer. In subsequent ELISA experiments, we detected the diagnostic efficacy of ARHGAP25 and INPP1 in the validation cohort and determined the serum expression level of breast cancer. After the analysis of ELISA results, we observed that the expression levels of these two proteins in the blood of early and advanced breast cancer patients were significantly lower than those in the healthy control group (p < 0.0001), which was consistent with the results obtained by Olink. To our knowledge, this is the first study to report an association between INPP1 and breast cancer, to report serum downregulation of INPP1, and to confirm serum downregulation of ARHGAP25. This study further found that the expression levels of ARHGAP25 and INPP1 in breast cancer HER-2 overexpression type were significantly downregulated (p < 0.0001). This inverse correlation suggests that HER-2 overexpression is associated with the potential suppression of these proteins, possibly involving their related pathways. In addition, the serum level of ARHGAP25 was significantly negatively correlated with the Ki-67 index of tumor tissue (r=−0.5501, p < 0.0001), indicating that ARHGAP25 may play an anticancer role by inhibiting the progression of the cell cycle.

While traditional markers CA15–3 and CEA demonstrate moderate performance in advanced breast cancer (AUC 0.75–0.85), their utility for early diagnosis remains limited (AUC<0.65). The AUC of INPP1/ARHGAP25 combined diagnostic model in early breast cancer was 0.7598, which was >15% higher than the existing markers, providing a new strategy for non-invasive early screening. Notably, this combined model demonstrated even more powerful diagnostic performance in advanced breast cancer (stage III-IV), achieving an AUC of 0.9483 with high sensitivity (81.1%) and specificity (98.1%).In addition, the expression level of INPP1 and ARHGAP25 in advanced breast cancer was significantly lower than that in early breast cancer (P = 0.454), suggesting that INPP1/ARHGAP25 can be used as a potential indicator of the severity of breast cancer.

There are still some limitations in this study, such as the small sample size, which can be verified in the future with a multicenter cohort. Finally, this study has not yet been involved in the mechanism research, and the molecular mechanism can be verified and resolved by constructing a gene knockout mouse model in the future. It can also be combined with multi-omics research to improve the performance of the model and solve the problems of early diagnosis and dynamic monitoring of clinical breast cancer.

While the present study provides robust evidence for the diagnostic value of the INPP1/ARHGAP25 serum protein panel, further multi-omics integration could offer deeper insights into its biological underpinnings. Future work will focus on interrogating large-scale genomic datasets (e.g., GWAS catalogs) to determine whether genetic variants regulating the circulating levels of these proteins (pQTLs) are also associated with breast cancer susceptibility. Additionally, leveraging transcriptomic data from repositories such as TCGA will help elucidate whether tumor tissue expression of these genes correlates with serum levels and patient prognosis. These analyses have the potential to bridge the gap between correlation and causation, positioning INPP1 and ARHGAP25 not only as diagnostic biomarkers but also as key players in breast cancer pathogenesis.

## Conclusion

This study combined Olink proteomics and a multi-protein combination strategy to determine the diagnostic efficacy of INPP1 and ARHGAP25 in breast cancer by subsequent ELISA verification. In this study, we found the association between INPP1 and breast cancer for the first time, and detected the protein expression level of ARHGAP25 in breast cancer serum for the first time. The combination of these two proteins significantly improves the efficiency of early breast cancer diagnosis.

## Declarations

We appreciate the helpful assistance and perceptive remarks from every member of our laboratories.

## Funding

This work was supported by the Shanghai Institute of Biomedical Technology and Tianjin Keweichuang Biotechnology Co., Ltd.

## Ethics approval and consent to participate

All experiments were conducted in accordance with the study protocol approved by the Ethics Committee, and all patients signed the informed consent form with the registration number of the approved study protocol KY-2022–043.

## Consent for publication

All authors approved the manuscript for publication.

## Availability of data and materials

Data will be made available on reasonable request.

## Code availability

Not applicable.

## CRediT authorship contribution statement

**Jingchun Huang:** Writing – original draft, Investigation, Data curation. **Lixin Gao:** Writing – original draft, Validation. **Liliia Glazutdinova:** Investigation. **Xuyao Ji:** Investigation. **Jing Zhang:** Data curation. **Youmei Lu:** Validation. **Siyao Zhang:** Validation. **Yu Liu:** Writing – review & editing, Resources.

## Declaration of competing interest

All authors approve this submission and declare no conflicts of interest (COI form attached) The study protocol was approved by the Ethics Committee of the Fourth Affiliated Hospital of Harbin Medical University, China (approval No. KY-2022-043). All participants provided written informed consent.
